# Monitoring of Chilika Lake mouth dynamics and quantifying rate of shoreline change using 30 m multi-temporal Landsat data

**DOI:** 10.1016/j.dib.2018.12.082

**Published:** 2018-12-29

**Authors:** Vivek G, Santonu Goswami, R.N. Samal, S.B. Choudhury

**Affiliations:** a Ocean Colour Applications and Measurement Division, Earth and Climate Sciences, National Remote Sensing Centre, Dept. of Space, Hyderabad 500037, India; b Chilika Development Authority, Dept. of Forest and Environment, Govt. of Odisha, 751014, India

## Abstract

Coastal erosion is one of the major and serious concerns for coastal communities residing in the low lying areas, especially near to estuary delta regions. These regions see lots of anthropogenic activities such as economic development, infrastructure and human settlement especially in rapidly developing countries such as India. Shoreline change is a natural process that occurs in coastal areas. But due to the stresses happening in the coast because of anthropogenic activities, understanding how shorelines change over time is important for sustainable management of coast. A crucial aspect of shoreline change monitoring is to identify the location and change over time which can be achieved by developing monitoring strategies using satellite remote sensing data. Performing shoreline change analysis using long term satellite records will help us to understand how shorelines respond to coastal development over time. In the present study we investigate shoreline erosion and accretion rate using three temporal Landsat scenes acquired over a thirty year period for the years 1988, 2000 and 2017. Digital Shoreline Change Analysis System (DSAS) an extension of ArcGIS software was used to compute rate of change statistics by calculating End Point Rate (EPR) values. We observed that Chilika coast is experiencing both erosion and accretion process with very high erosion rate of −13.6 m/yr and accretion of 13.5 m/yr, at Chilika Lake mouth. The average erosion and accretion rate of −1.13 m/yr and 1.41 m/yr were recorded for the study region.

**Specifications table**

**Table:** 

Subject area	*Remote Sensing*
More specific subject area	*Remote Sensing and GIS*
Type of data	*Satellite image, tables, figures*
How data was acquired	*Downloaded from USGS website*
Data format	*Analyzed*
Experimental factors	*Shoreline extracted and change rate calculated using ArcGIS software and DSAS tool*
Experimental features	*Erosion and accretion rate calculated to understand lake mouth dynamics for the period 1988–2017*
Data source location	*Chilika, North–East coast of Odisha, India.*
Data accessibility	*Data are available with this article*
Related research article	*Santonu Goswami, Vivek G., S.B Choudhury, “Three Decades of Landcover Change in Chilika and its Neighbourhood Area using 30 m Landsat Data”, 38th Asian Conference of Remote Sensing, Delhi.*
	doi:10.13140/RG.2.2.16408.29448

**Value of the data**

• Data are georeferenced and digitized for any future studies.
• Data can be used to understand dynamics of lake mouth over a thirty year period by quantifying erosion and accretion process during 1988–2017.
• The data produced here will be useful for coastal communities, researchers, policymakers and stakeholders working on coastal ecosystems.

## 1 Data

**Fig. 1:**
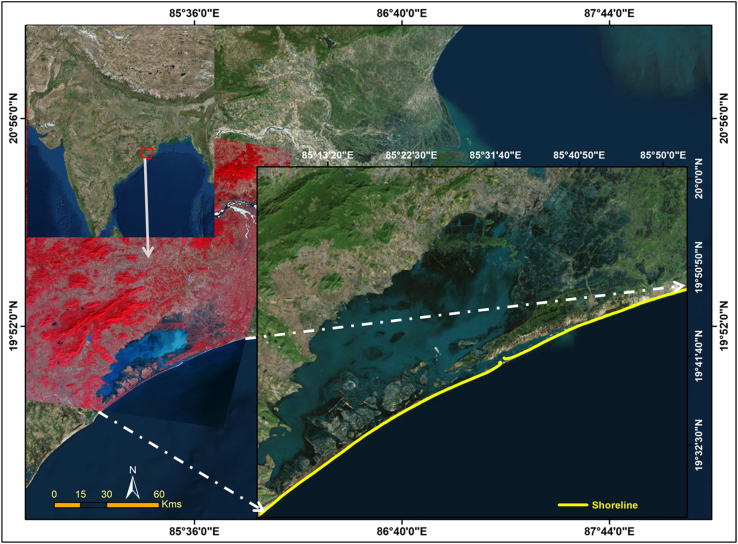
Location map of the study area.

The data presented in this study will help us to understand the dynamics of lake mouth as well as the erosion and accretion process happening in the coast for past four decades. Fig. 1 shows the location of study area situated in north east coast of India with a coastal stretch of 120 km. Shoreline change assessment was carried out to understand erosion and accretion process, by taking three temporal year׳s satellite imageries of Landsat for the period of 1988, 2000 and 2017. Details of data used in this study are given in Table 1. The Shorelines were extracted for these years which cover 29 years period and rate of change statistics was computed by calculating End Point Rate (EPR). The Changes in lake mouth can be seen clearly in satellite images given in Fig. 6 for different period of time. All the data were downloaded free of charge from USGS earth explorer (https://earthexplorer.usgs.gov/).

**Table 1:** Details of data used for study.

**Satellite**	**Sensor**	**Date of acquisition**	**Resolution**	**Path/row**	**No. of bands**
Landsat 4–5	TM	07 Jan 1988	30 m	140/46	7
Landsat 7	ETM+	17 Dec 2000	30 m	140/46	8
Landsat 8	OLI/TIRS	06 Jan 2017	30 m	140/46	11

## 2 Experimental design, materials, and methods

### 2.1 Methodology

**Fig. 2:**
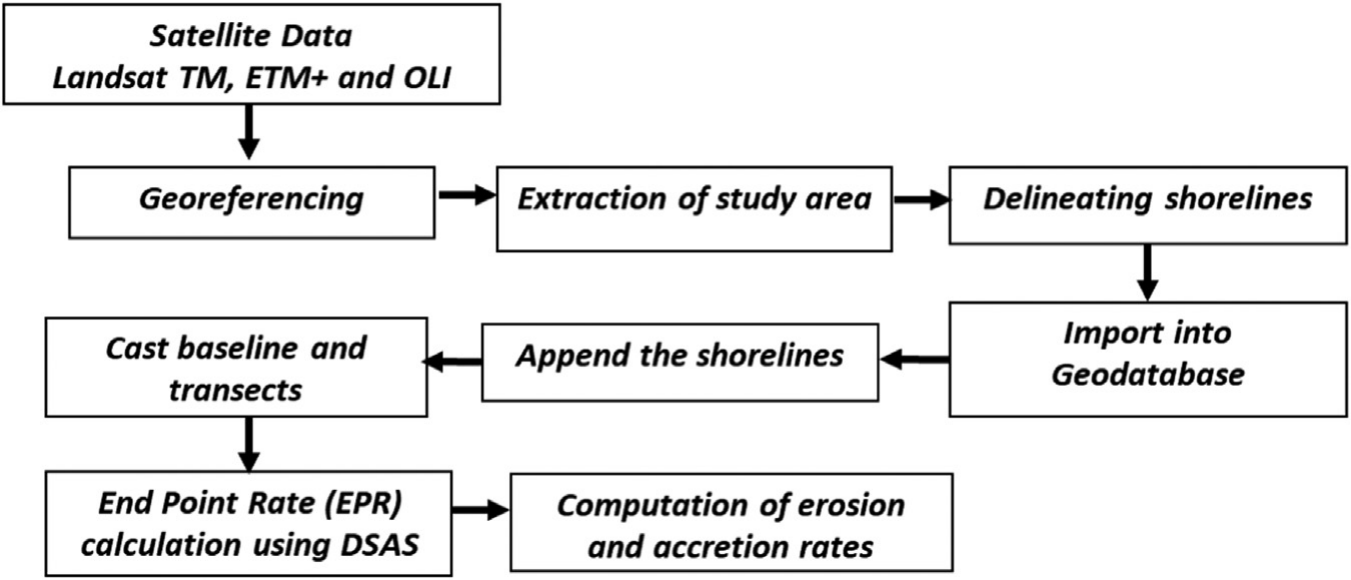
Methodology for estimating shoreline erosion and accretion rate.

The current study site Chilika, located along the north east coast of Odisha with a coastal stretch of 120 km. Flowchart given in Fig. 2 depicts overall workflow to identify erosion and accretion process over study region. Multi-resolution and multi-temporal Landsat data were obtained from USGS for the period of 07 Jan 1988, 17 Dec 2000 and 06 Jan 2017. Shorelines were extracted by digitizing manually from these satellite imageries by identifying wet and dry boundary line. Extracted shoreline will be in vector format which will be given as input for Digital Shoreline Analysis System (DSAS) toolbar, an extension of ArcGIS software [1].

**Fig. 3:**
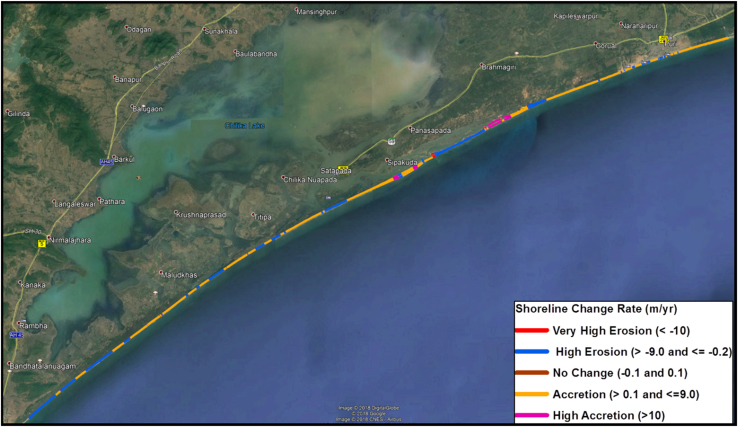
Extracted shoreline was overlaid on Google earth image and classified into five classes based on EPR values.

**Fig. 4:**
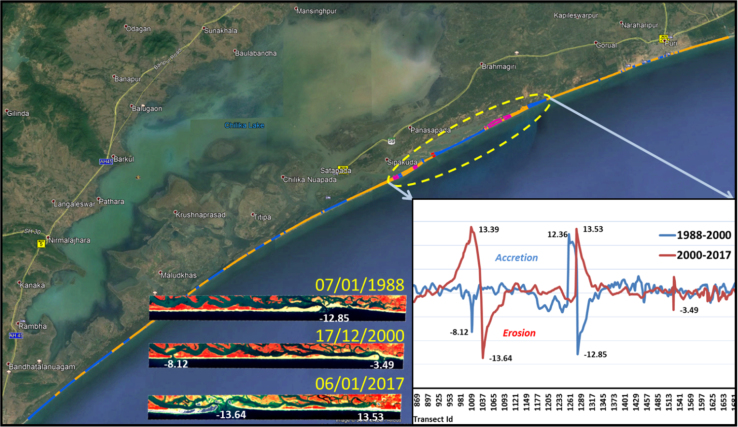
Map showing erosion and accretion process at Chilika Lake mouth with EPR values along with corresponding satellite images of lake mouth.

**Fig. 5:**
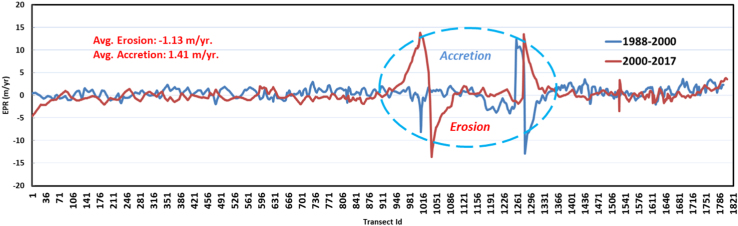
Graph depicts erosion and accretion process over Chilika coast and at lake mouth highlighted with dashed line for a period of 1988–2000 and 2000–2017.

**Fig. 6:**
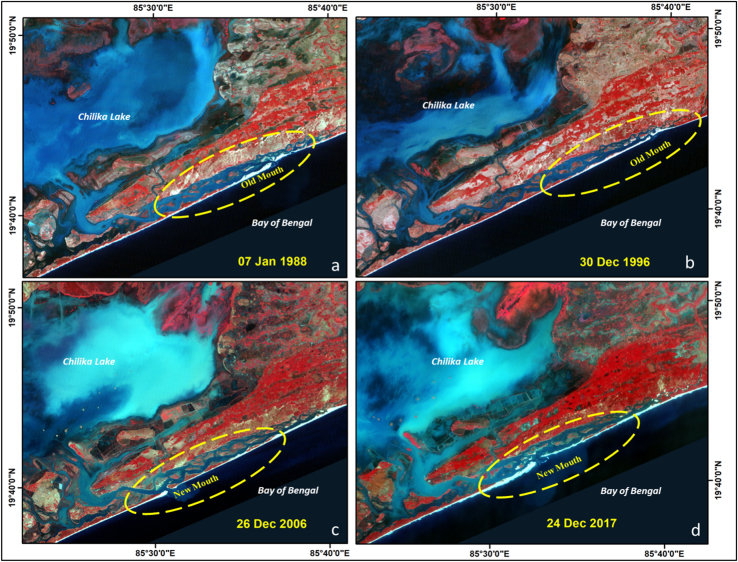
Satellite imageries show the dynamics of Lake Mouth for different periods. (a) Jan 1988 Landsat TM image. (b) Dec 1996 Landsat TM image. (c) Dec 2006 Landsat TM image. (d) Dec 2017 Landsat OLI image.

Totally 1861 transects were generated with 25 m spacing and the length of transects was 500 m (Table 2). Rate of change statistics was computed by calculating End Point Rate (EPR) and values obtained from EPR will be classified into five categories which are: very high erosion, high erosion, no change, accretion and high accretion (Fig. 3). An average Erosion/Accretion of −1.13 m/yr and 1.41 m/yr was recorded, while the highest Erosion/Accretion with recorded value of −13.63 m/yr and 13.9 m/yr was observed at Chilika Lake mouth ( Figs. 4 and 5). The migration and formation of new mouth can be seen clearly on satellite image given in Fig. 6 for the different periods of time.

**Table 2:** Transect details for study area.

No. of transects created	Length of transects (m)	Transects spacing (m)	Cast direction
1861	500	25	Onshore
